# CA 15-3 prognostic biomarker in SARS-CoV-2 pneumonia

**DOI:** 10.1038/s41598-022-10726-7

**Published:** 2022-04-25

**Authors:** José Antonio Ros-Lucas, Domingo Andrés Pascual-Figal, José Antonio Noguera-Velasco, Álvaro Hernández-Vicente, Iria Cebreiros-López, María Arnaldos-Carrillo, Isabel M. Martínez-Ardil, Elisa García-Vázquez, Mario Aparicio-Vicente, Elena Solana-Martínez, Sheyla Yolany Ruiz-Martínez, Laura Fernández-Mula, Rubén Andujar-Espinosa, Beatriz Fernández-Suarez, Maria Dolores Sánchez-Caro, Carlos Peñalver-Mellado, Francisco José Ruiz-López

**Affiliations:** 1 Pneumology Service, Clinical University Hospital Virgen de La Arrixaca, Murcia, Spain; 2 IMIB- Arrixaca, Murcia, Spain; 3 Catholic University Murcia (UCAM), Murcia, Spain; 4 Cardiology Service, Clinical University Hospital Virgen de La Arrixaca, , Murcia, Spain; 5 University of Murcia (UMU), Murcia, Spain; 6 National Center for Cardiovascular Research (CNIC), Madrid, Spain; 7 CIBER Cardiovascular, Murcia, Spain; 8 Clinical Laboratory Service, Clinical University Hospital Virgen de La Arrixaca, Murcia, Spain; 9 Family and Community Medicine, Murcia Health Service, Murcia, Spain; 10 Internal Medicine, Infectious Diseases Section, Clinical University Hospital Virgen de La Arrixaca, Murcia, Spain

**Keywords:** Prognostic markers, Infectious diseases

## Abstract

The severity of lung involvement is the main prognostic factor in severe acute respiratory syndrome coronavirus 2 (SARS-CoV-2) infection. Carbohydrate antigen 15-3 (CA 15-3), a marker of lung damage and fibrosis, could help predict the prognosis of SARS-CoV-2 pneumonia. This was a retrospective and observational study. CA 15-3 was analyzed in the blood samples of patients consecutively admitted for SARS-CoV-2 pneumonia and whose blood samples were available in the biobank. Other prognostic markers were also measured (interleukin 6 [IL6], C-reactive protein [CRP], D-dimer, troponin T, and NT-ProBNP). The occurrence of in-hospital complications was registered, including death, the need for medical intensive care, and oxygen therapy at discharge. In this study, 539 patients were recruited (54.9% men, mean age: 59.6 ± 16.4 years). At admission, the mean concentrations of CA 15-3 was 20.5 ± 15.8 U/mL, and the concentration was correlated with male sex, older age, and other severity markers of coronavirus disease of 2019 (COVID-19) (IL6, CRP, D-dimer, troponine T, and NT-ProBNP). CA 15-3 levels were higher in patients who died (n = 56, 10.4%) (35.33 ± 30.45 vs. 18.8 ± 12.11, *p* < 0.001), who required intensive medical support (n = 78, 14.4%; 31.17 ± 27.83 vs. 18.68 ± 11.83; *p* < 0.001), and who were discharged with supplemental oxygen (n = 64, 13.3%; 22.65 ± 14.41 vs. 18.2 ± 11.7; *p* = 0.011). Elevated CA 15-3 levels (above 34.5 U/mL) were a strong predictor of a complicated in-hospital course, in terms of a higher risk of death (adjusted odds ratio [OR] 3.74, 95% confidence interval [CI]: 1.22–11.9, *p* = 0.022) and need for intensive care (adjusted OR 4.56, 95% CI: 1.37–15.8) after adjusting for all other risk factors. The degree of lung damage and fibrosis evaluated in terms of CA 15-3 concentrations may allow early identification of the increased risk of complications in patients with SARS-CoV-2 pneumonia.

## Introduction

The clinical spectrum of severe acute respiratory syndrome coronavirus 2 (SARS-CoV-2) infection is broad, ranging from asymptomatic infection and mild upper respiratory infection to severe pneumonia with respiratory failure and death^1^. Although knowledge regarding the clinical behavior of SARS-CoV-2 pneumonia is gradually expanding, the various influences that make some patients present a worse evolution still remain unidentified. Some factors have been determined to be associated with a worse prognosis, such as age (being older) or sex (being male), and some prognostic scales have been established^2,3^, that could provide support in assessments, although their usefulness is not yet clear.

The pulmonary epithelium constitutes the primary line of defense against viral respiratory infections^4^. The initial lung lesion that develops either because of the virus alone or the corresponding inflammatory response affects the alveolar epithelium and the capillary endothelium^5^. Mucus plays a fundamental role in protecting the respiratory tract against microbial infections^6^, with its secretion increasing when the lung epithelium is damaged^7^. Mucus is the first site of contact for respiratory microbes, including SARS-CoV-2^8^, where they are trapped; their elimination is facilitated through the drainage of secretion by the mucociliary system.

Acute respiratory distress syndrome (ARDS) is triggered in the most severe forms of coronavirus disease of 2019 (COVID-19) pneumonia. In autopsies of patients who died because of ARDS caused by COVID-19, exudative and proliferative phases of diffuse alveolar damage were observed, with hyaline membranes, hyperplasia of atypical pneumocytes, alveolar hemorrhages, infarcts, and endothelial damage, as well as capillary congestion and microthrombi^9^. Unlike ARDS stemming from other causes, the involvement of the vasculature is higher with a COVID-19 diagnosis, including thrombosis, endothelial cell injury, vascular dilation, and aberrant angiogenesis^10^. When the vascular endothelium is infected, its capillary permeability increases and it further acquires a proinflammatory phenotype with the production of cytokines (interleukin 1 [IL1], tumor necrosis factor, and IL-6)^11^.

Mucins 1, 4 and 16 (MUC1, MUC4, and MUC16) are the three major transmembrane airway mucins that prevent microbial invasion, act as releasable decoy receptors, and activate intracellular signal transduction pathways. Mucin expression and glycosylation depend on the inflammatory state of the respiratory tract and are directly regulated by proinflammatory cytokines and microbial ligands^6^. Elevated MUC1 levels have been observed in the sputum and tracheal aspirates of patients with COVID-19^12^. MUC1 is a large glycoprotein that acts as a membrane receptor and consists of three domains, an extracellular domain, a single transmembrane region, and a cytoplasmic tail (CT). The bioactive CT (MUC1-CT) has been shown to have anti-inflammatory effects in respiratory infections, and by interacting with some effectors, it intervenes in the carcinogenic and fibrotic processes of the lung, which makes it an interesting biomarker of pulmonary processes^13^. The Krebs von den Lungen 6 (KL-6) marker and carbohydrate antigen 15-3 (CA 15-3) are soluble subunits of the N-terminal region of MUC1^13^.

The KL-6 is a prognostic marker of interstitial lung disease^14,15^, and CA 15-3 is an alternative marker for KL-6^16^. Studies have shown an elevation of KL-6 and CA 15-3 markers in patients with SARS-CoV-2 pneumonia^17–19^. Unlike KL-6, CA 15-3 is more measurable in clinical practice because it is used as a tumor marker. Therefore, this study evaluated the efficacy of the CA 15-3 concentration as a marker of fibrosis and severity of lung damage, as well as its potential application in risk stratification in patients with COVID-19 pneumonia upon admission.

## Methodology

### Study population

An observational and retrospective study was performed in patients consecutively hospitalized for SARS-CoV-2 infection, confirmed by polymerase chain reaction (PCR) testing, with pulmonary infiltrates on chest-X-ray, between April 2020 and March 2021. According to Arrixaca Hospital´s COVID-19 protocol, a blood sample was prospectively obtained from these patients at the time of hospital admission, and this was deposited in the biobank. All patients provided signed informed consent for such storing of samples for research. The study was carried out following all the relevant guidelines and regulations and was approved by the ethics committee of the Virgen de la Arrixaca hospital in Murcia, Spain.

### Clinical variables and events

The characteristics of the patients were collected upon admission and during hospitalization. These variables included previously established clinical and analytical risk markers as well as the World Health Organization (WHO) COVID Ordinal Outcomes Scale (Table 4S)^20^. During hospitalization, the events of the study recorded included death, the need for intensive medical care (high flow, non-invasive mechanical ventilation, invasive mechanical ventilation, or extracorporeal membrane oxygenation), attainment of the highest WHO score level, and the need for oxygen therapy at discharge.

### Analytical methods

Blood samples were collected at the time of admission in vacuum tubes using lithium heparin as the anticoagulant for biochemistry tests, EDTA K3 for hemograms, and citrate for coagulation tests. The plasma obtained was used for panel determination. A biochemical analysis was performed for renal function, urea, creatinine, and ions (sodium, potassium, and chlorine) using a Cobas 702 analyzer. Ferritin and C-reactive protein (CRP) were identified by immunoturbidimetry via the same platform, and troponin T, NT-ProBNP, IL-6, and CA 15-3 were determined by electrochemiluminescence Immunoassay (ECLIA) on a Cobas e 801 analyzer. All tests were performed on a Modular Cobas 8000 system from Roche Diagnostics. D-dimer determinations were carried out on citrated plasma with a Werfen ACL 350 analyzer. Furthermore, the Sysmex XN 4000i hematology analyzer was used for blood analysis.

The CA 15-3 test was performed on the peripheral blood. A sandwich-type assay was performed in which the sample antigen, a biotinylated monoclonal antibody specific for CA 15-3, and a monoclonal antibody specific for CA 15-3 labeled with a ruthenium complex reacted to form a sandwich complex. After the addition of streptavidin-coated microparticles, the complex bound to the solid phase through an interaction between biotin and streptavidin. The microparticles were magnetically captured on the electrode surface, and the application of a voltage to the electrode induced a chemiluminescent emission proportional to the amount of CA 15-3 in the sample.

### Statistics

The mean with standard deviation (mean ± SD) and medians with interquartile range (median [IQR]) for the characteristics at baseline and end points were calculated when normally distributed or skewed, respectively. Frequencies with percentages (*n* (%)) were calculated for categorical variables. To estimate the differences according to quartiles of CA 15-3 levels, because there were more than two groups, analysis of variance (ANOVA) and Kruskal Wallis tests were used in the case of continuous variables. For categorical variables, Chi-square tests (Fisher's exact tests) were used for the characteristics table, and the asymptotic linear-by-linear association tests were used for the end-points table (to emphasize the linear character of the marker). Correlations between markers were estimated with Spearman's rho coefficients.

The new marker was categorized into levels of risk to analyze its predictive power. To calculate them, Peirce and Cornell's method of stratum-specific likelihood ratios was used. Starting with five strata with uniformly distributed death events, the method provided three strata. Logistic regression models with the stratified marker were adjusted to analyze death, need for intensive medical support, and requirement of supplemental oxygen at discharged. All significant variables in the unadjusted models were used as covariates. Logarithmic transformation was applied to some variables to achieve the linearity required for the models. All analyses were performed using the statistical software R, version 4.1, and SPSS 21.

### Ethics approval and consent to participate:

All patients signed an informed consent upon admission to save blood samples in the hospital biobank. The study was approved by the hospital's ethics committee. Code of the committee of the Hospital: 2021-3-15 HCUVA.

### Consent for publication

The article does not contain personal data that require consent.

## Results

### Study population

We studied 539 patients who were hospitalized because of COVID-19. The population had a mean age of 59.6 years, with 31% being over 70 years old. Male patients represented 54.9% of the sample, and their mean age did not differ significantly from that of female patients (Table 1). Among comorbidities, arterial hypertension was the most frequent (44%), followed by diabetes mellitus (26%), chronic cardiovascular disease (17.1%), and pulmonary disease (16.3%).

**Table 1:** Distribution of the clinical characteristics at baseline by quartiles of CA15-3.

	Overall	CA15-3				*p*
		[1.95,10.8]	[10.8,17.1]	[17.1,25.3]	[25.3,190]	
N	539	129	131	130	130	
Age, years	59.57 ± 16.36	50.17 ± 14.66	59.12 ± 16.36	61.26 ± 15.46	68.64 ± 13.27	< 0.001
Male	296 (54.9)	52 (40.3)	71 (54.2)	76 (58.5)	86 (66.2)	< 0.001
**History**						
Respiratory disease	88 (16.3)	12 (9.3)	15 (11.5)	25 (19.2)	35 (26.9)	< 0.001
*Smoking*						0.016
No	394 (74.9)	101 (79.5)	99 (78.6)	99 (78.0)	81 (63.8)	
Yes	30 (5.7)	5 (3.9)	10 (7.9)	4 (3.1)	9 (7.1)	
Former	102 (19.4)	21 (16.5)	17 (13.5)	24 (18.9)	37 (29.1)	
Hypertension	237 (44.0)	32 (24.8)	60 (45.8)	67 (51.5)	71 (54.6)	< 0.001
Diabetes	140 (26.0)	29 (22.5)	29 (22.1)	36 (27.7)	38 (29.2)	0.443
Cardiovascular disease	92 (17.1)	9 (7.0)	19 (14.5)	22 (16.9)	38 (29.2)	< 0.001
History of cancer	46 (8.6)	4 (3.1)	6 (4.6)	10 (7.7)	23 (17.8)	< 0.001
**Characteristics at admission**						
Length of symptoms, days	6 [3, 9]	6 [3, 8]	6 [3, 8]	6 [3, 9.25]	6 [3, 10]	0.569
*Facility of admission*						0.001
Ward	499 (92.6)	126 (97.7)	123 (93.9)	121 (93.1)	110 (84.6)	
Intensive care	40 (7.4)	3 (2.3)	8 (6.1)	9 (6.9)	20 (15.4)	
WHO scale	3.73 ± 0.63	3.57 ± 0.54	3.72 ± 0.62	3.78 ± 0.65	3.89 ± 0.68	< 0.001
D-dimer, ng/mL	272 [177, 511]	216 [150, 318]	261 [167, 528]	301 [197, 517]	334 [224, 817]	< 0.001
Ferritine, ng/mL	457 [227, 993]	366 [158, 690]	474 [232, 970]	501 [245, 1008]	691 [292, 1411]	< 0.001
IL-6, pg/mL	38.4 [15.2, 69.3]	28.1 [12.5, 55.8]	39.2 [13.1, 70.8]	41.7 [17.9, 69.2]	43 [21.4, 82.2]	0.008
CRP, mg/dL	6.09 [2.62, 11.6]	4.24 [1.83, 9.05]	7.28 [3.04, 13.18]	6.34 [2.8, 11.4]	6.54 [3.29, 12.9]	0.011
TnT-hs, pg/mL	8 [5, 16]	5 [3.62, 7]	7.03 [5, 12.8]	9 [6, 18.8]	15 [8, 26.8]	< 0.001
NT-proBNP, pg/mL	124 [40, 405]	67 [24.1, 164]	100 [35.9, 279]	154 [54.7, 342]	270 [80.8, 1197]	< 0.001

### CA 15-3 concentrations

At admission, the concentration of CA 15-3 had a mean of 20.5 ± 15.8 U/mL. Table 1 shows the distribution of the clinical characteristics at admission according to the quartiles of CA 15-3. Significant interquartile differences were observed between CA 15-3 concentrations, male gender, and older age. They were also observed between smoking, arterial hypertension, chronic diseases (including cardiovascular and pulmonary diseases), and a history of neoplasia. High levels of CA 15-3 have higher concentrations of inflammatory and cardiac biomarkers (supplemental material provided in Table 1S). The prevalence of diabetes and the length of the symptoms did not differ across the quartiles of CA 15-3.

### CA 15-3 concentrations and in-hospital evolution

**Figure 1:**
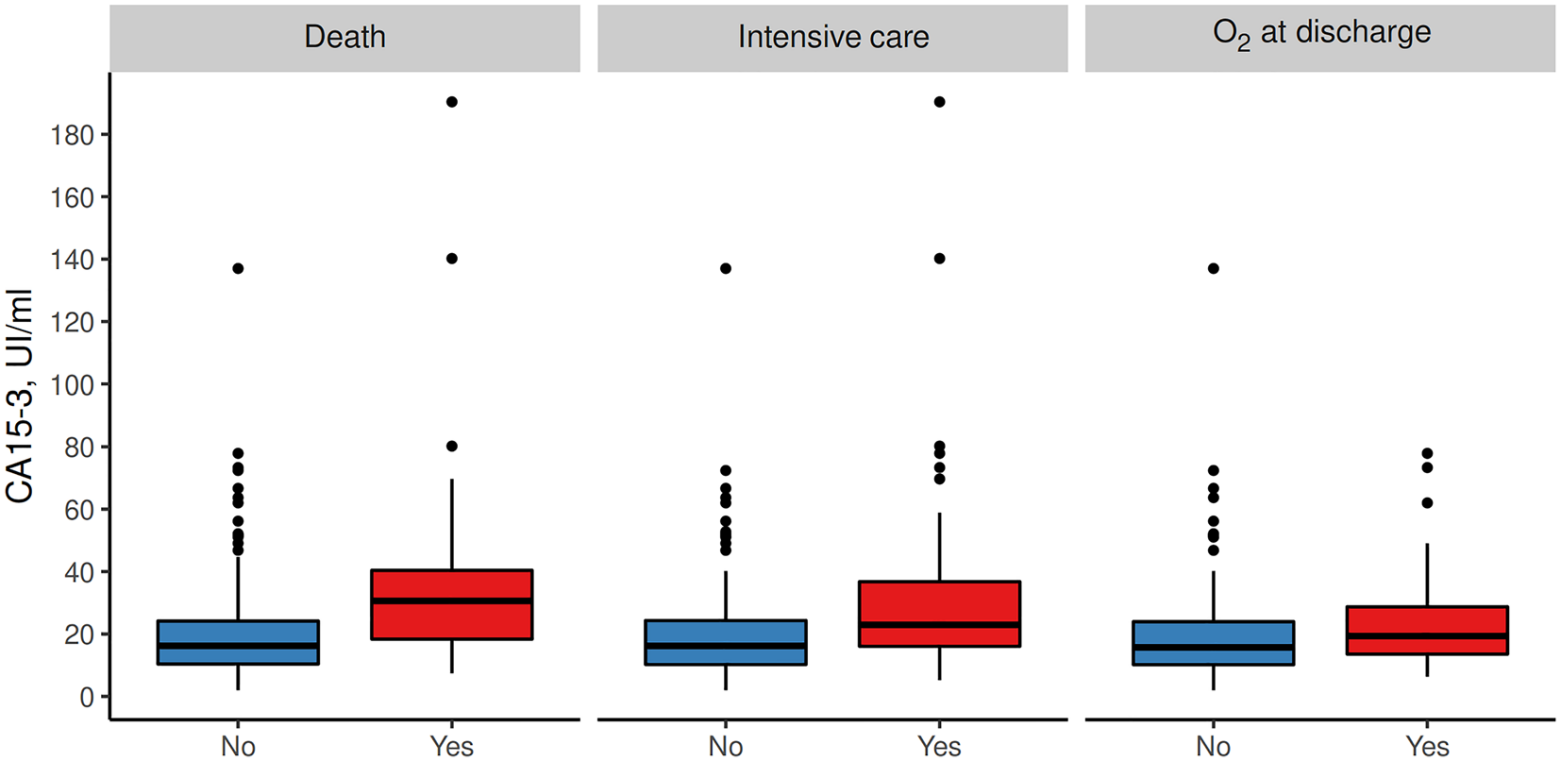
Distribution of CA 15-3 based on adverse events.

As shown in Table 2, all adverse events significantly increased across the quartiles of CA 15-3. During the in-hospital evolution, 56 patients died (10.4%), 78 needed to be admitted to intensive care facilities (14.4%), and 64 (13.3%) required oxygen therapy at discharge. As shown in Fig. 1, the concentrations of CA 15-3 at admission were higher in patients who died (35.33 ± 30.45 vs. 18.8 ± 12.11, *p* < 0.001), who needed intensive medical support (31.17 ± 27.83 vs. 18.68 ± 11.83, *p* < 0.001), and who were discharged with supplemental oxygen (22.65 ± 14.41 vs. 18.2 ± 11.7, *p* = 0.011).

**Table 2:** Distribution of clinical end-points by quartiles of concentrations of CA 15-3.

	Overall	CA15-3				*p*
		[1.95,10.8]	[10.8,17.1]	[17.1,25.3]	[25.3,190]	
n	539	129	131	130	130	
Death	56 (10.4)	4 (3.1)	8 (6.1)	12 (9.2)	31 (23.8)	< 0.001
Intensive care	78 (14.5)	9 (7.0)	14 (10.9)	22 (16.9)	33 (25.4)	< 0.001
WHO maximal	4.09 ± 1.34	3.71 ± 0.89	4.02 ± 1.20	4.10 ± 1.25	4.62 ± 1.74	< 0.001
WHO at discharge	3.14 ± 1.72	2.73 ± 1.11	2.95 ± 1.43	3.06 ± 1.71	3.92 ± 2.26	< 0.001
Length of stay, days	6 [4, 10]	5 [3, 8.75]	8 [5, 11]	6 [4, 9]	7 [4, 11.5]	0.003
O2 at discharge	64 (13.3)	12 (9.6)	15 (12.2)	18 (15.5)	19 (19.2)	0.029

Among patients who required intensive care, 35 died (44,8%; 10 women and 25 men, *p* = 0.435). The deceased were older (68.37 ± 13 years vs. 62.52 ± 11.5 years; *p* = 0.031); no differences were observed between comorbidities or the presence or absence of previous pulmonary or cardiovascular disease or a history of tumor. The deceased had a higher level of CA 15-3 (41.2 ± 36.34 vs. 23.25 ± 14.75; *p* = 0.002); they also had significantly higher levels of CRP, D-dimer, troponin, IL6 and NT-ProBNP, but not ferritin.

### CA 15-3 as a predictor of adverse events

**Figure 2:**
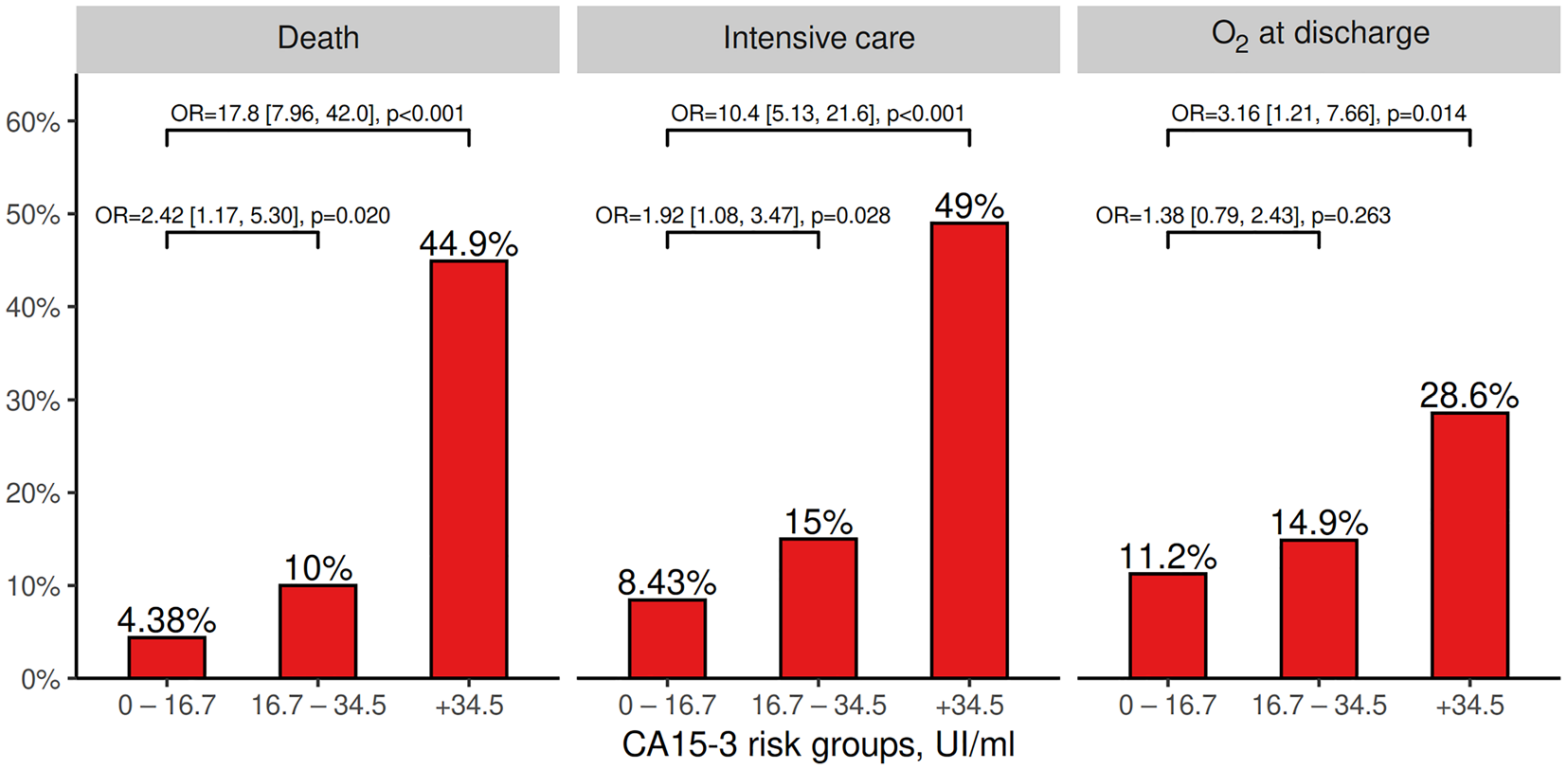
The rate of adverse events for each level of CA 15-3.

The receiver operating curve analysis showed an area under the curve of 0.752 (0.680–0.824) for the prediction of death and 0.688 (0.621–0.754) for the need for intensive medical care. An analysis of the optimal reference values revealed the following for prediction of death: < 16.7, 16.7–34.5, and > 34.5 U/mL (supplemental material available in Table 2S). Figure 2 shows the rate of adverse events at each level of CA 15-3 and the associated risk for each adverse end point.

Among the 49 patients with CA 15-3 > 34.5 U/mL at admission, the rates of adverse events during hospitalization were 45% for death, 49% for intensive care, and 29% for oxygen therapy at discharge. The corresponding odds ratio (OR) values were 17.8 (95% confidence interval [CI]: 7.96–42), 10.4 (95% CI: 5.13–21.6), and 3.16 (95% CI: 1.21–7.66%) for death, intensive care, and oxygen therapy at discharge, respectively (Fig. 2). The results of the adjusted analysis of predictors for adverse events are presented in Table 3 (univariate analysis in supplemental material, Table 3S). After adjustment for all other risk factors, an elevated CA 15-3 (above 34.5 U/mL) was a strong predictor of death or the need for intensive care.

**Table 3:** Multivariable adjusted analysis of predictors at baseline of complications during in-hospital evolution of patients with COVID19.

	Death		Intensive care		O_2_ at discharge	
	OR (95% CI)	*p*	OR (95% CI)	*p*	OR (95% CI)	*p*
Age, per 10 years	1.49 (1.01, 2.27)	0.053	0.85 (0.59, 1.24)	0.396	1.21 (0.85, 1.76)	0.298
Male	1.30 (0.49, 3.58)	0.599	2.15 (0.86, 5.67)	0.108	4.61 (1.79, 13.0)	0.002
Respiratory disease	1.56 (0.58, 4.07)	0.365	1.14 (0.42, 2.98)	0.787	2.30 (0.94, 5.56)	0.065
Smoking history	0.52 (0.17, 1.45)	0.224	0.65 (0.24, 1.65)	0.378	0.74 (0.28, 1.86)	0.526
Hypertension	0.91 (0.36, 2.36)	0.845	0.93 (0.37, 2.30)	0.877	0.74 (0.31, 1.72)	0.486
Diabetes	0.70 (0.28, 1.67)	0.433	0.48 (0.17, 1.26)	0.153	0.70 (0.28, 1.64)	0.420
Cardiovascular disease	0.48 (0.17, 1.29)	0.156	0.27 (0.07, 0.93)	0.048	0.96 (0.34, 2.58)	0.937
History of cancer	2.54 (0.90, 7.01)	0.073	1.88 (0.55, 6.07)	0.299	0.78 (0.17, 3.06)	0.734
WHO scale, per unit	4.21 (2.14, 8.92)	< 0.001	63.1 (22.1, 227)	< 0.001	33.1 (11.4, 126)	< 0.001
D-dimer, log10	1.97 (0.86, 4.38)	0.098	0.86 (0.30, 2.42)	0.780	0.85 (0.31, 2.18)	0.740
Ferritin, log10	2.31 (0.91, 6.20)	0.086	4.57 (1.64, 13.7)	0.005	1.49 (0.56, 3.97)	0.426
IL-6, log10	2.17 (0.93, 5.43)	0.083	1.50 (0.64, 3.79)	0.367	1.82 (0.67, 5.45)	0.258
CRP, log10	0.55 (0.17, 1.80)	0.312	0.43 (0.13, 1.37)	0.153	1.53 (0.49, 4.91)	0.465
TnThs, log10	10.2 (2.48, 44.4)	0.001	2.41 (0.58, 9.94)	0.220	5.10 (1.22, 21.2)	0.024
NT-proBNP, log10	1.06 (0.54, 2.04)	0.871	1.34 (0.66, 2.72)	0.419	1.27 (0.67, 2.44)	0.461
**CA15-3**						
0–16.7, reference	–	–	–	–	–	–
16.7–34.5	1.38 (0.52, 3.86)	0.523	1.01 (0.41, 2.49)	0.977	0.54 (0.24, 1.19)	0.130
> 34.5	3.74 (1.22, 11.9)	0.022	4.56 (1.37, 15.8)	0.014	0.75 (0.19, 2.70)	0.666

## Discussion

Lung involvement in SARS-CoV-2 infection helps determine the disease prognosis. The determination of markers that help in the early identification of the degree of pulmonary involvement and therefore of damage to the alveolar epithelium can help in early therapeutic decision making^21^. Early treatment is a prognostic factor, especially for patients with serious conditions^22^.

Patients with SARS-CoV-2 infection can present a wide range of symptoms, from minimal respiratory symptoms to the appearance of severe ARDS, requiring ventilatory support and even causing death in some cases. These differences are defined by the host´s capacity to limit infection; without such capacity, the alveolar epithelium can be significantly destroyed, and this can trigger an inflammatory response with a cytokine storm^4^. The initial lesion that occurs in the lung because of either viral involvement or the inflammatory response affects the alveolar epithelium and the capillary endothelium, with the appearance of interstitial edema and fluid leakage into the alveolus^5^. At the level of the alveolar epithelium, the virus mainly affects type 2 pneumocytes, altering the repair mechanisms of the alveolar epithelium and the production of surfactants, further favoring the production of cytokines^23^, the appearance of ARDS in adults^24^, and the development of residual fibrotic processes^25^.

Our study attempts to assess whether CA 15-3 measured at hospital admission can predict the prognosis of SARS-CoV-2 pneumonia. The KL6 marker and CA 15-3 are soluble subunits of the N-terminal region of MUC1, a mucin expressed in the lower respiratory tract and the tracheal, bronchial, and alveolar epithelial cells and elevated in the mucus of patients with COVID-19^26^. Studies have already shown that the KL-6 concentration is higher in the most severe cases of COVID-19 pneumonia^17,17^, and it could be used as an indicator of damage to the alveolar epithelium^27^, because this marker is increased in the lesions of the alveolar epithelium, in the regeneration processes, and in fibrosis^28^. Thus, the KL-6 concentration can be used to identify patients with the worst prognosis^17^ or residual fibrosis^29^. In our study, we proposed using CA 15-3 on admission as a marker of lung damage and fibrosis because it is a more generalizable and common marker than KL6, with which it is correlated^16^, and because CA 15-3 increases in COVID-19^30^.

Our results indicate that CA 15-3 is elevated in patients with worse prognosis, correlates with severity measured on the WHO Scale, is higher in those requiring intensive care, and is also higher in patients who die. Similar results have been published recently, in which higher levels of CA 15-3 have been observed in patients admitted to intensive care compared with those admitted to the ward, and it has been correlated with the degree of fibrosis measured with the computer tomography^19^ . Unlike this study, in our study, CA 15-3 was measured at the time of admission, relating to severity and the possibility of worsening health status and risk of death, especially among those requiring intensive care.

As observed in other studies, age, sex, and comorbidities are also poor prognostic factors in SARS-CoV-2 pneumonia^31^. Age is related to the response to infection, the reparative capacity of the alveolar epithelium, and the risk of residual fibrosis^32^. Older patients and men have been linked to a higher probability of mortality^33,33^; in accordance with these findings, in our study, the deceased were older than the survivors, and there was a higher proportion of men who require intensive care. Differences in sex between the deceased and survivors were ignored. Comorbidities have also been described in other studies as poor prognostic factors^35,36^; similarly, in our study, patients with hypertension; diabetes; or previous pulmonary, cardiovascular, or tumor pathology presented a worse prognosis.

The cytokine storm occurs in the exudative phase of ARDS, with the participation of the alveolar epithelium and vascular endothelium^4^. In our study, CA 15-3 correlated with both markers of inflammation (ferritin, CRP, and IL6, as well as thrombosis measured by D-dimer)^37^, and with markers of cardiac involvement (troponin T and NT-ProBNP)^31^, which are biomarkers associated with severe SARS-CoV-2 pneumonia^38,39^ and which were also higher in our subjects who required intensive care and those who died.

In addition to innate and acquired immunity to stop the infection, an accurate capacity to repair the damaged alveolar epithelium is necessary to ensure effective and comprehensive recovery from COVID-19 pneumonia^40^. The reparative processes of the lungs occur in the proliferative phase of ARDS, and if not produced correctly, there will be an increase in lung stiffness with the development of mechanisms of pulmonary fibrosis. This is defined as the fibrotic phase of ARDS and is correlated with mortality and the need for mechanical ventilation^4^. In the lung, greater inflammation and involvement of the pulmonary epithelium activate profibrotic processes, which are related to the degree of residual fibrosis^41^. In COVID-19 patients, elevated fibrosis markers, such as hyaluronic acid or type III procollagen, are indicators of poor prognosis^33^. MUC1 and CA 15-3 are also markers of pulmonary fibrotic processes^42^, and their elevation is related to a greater degree of pulmonary involvement and a worse prognosis^43^. In our study, patients who required oxygen therapy at discharge exhibited a higher level of CA 15-3 upon admission, which could be related to greater pulmonary damage and fibrosis^19,23^.

Other factors that have been related to the need for oxygen therapy at discharge are age, a history of pulmonary or cardiovascular diseases, and comorbidities, such as hypertension and diabetes. Age is not only related to increased severity but also to a higher risk of residual fibrosis because it affects the profibrotic potential of the pulmonary fibroblasts^25^ and the reparative capacity of the pulmonary epithelium. The presence of comorbidities, such as hypertension and diabetes, are related to metabolic syndrome, which has been associated with an increased risk of developing fibrosis^25^. Diabetes is associated with a lower production of surfactants at the alveolar level, which may facilitate the development of ARDS^44^.

Further studies are required to establish the role of mucins in SARS-CoV-2 infection. Elevated levels have been observed in the secretions of patients with COVID-19 and could be related to greater severity, as discussed in our study. MUC1 could have a protective effect on certain infections, including respiratory infections, especially in the inflammation resolution phase, and it could affect the evolution of other respiratory diseases, such as rhinitis, asthma, chronic obstructive pulmonary disease, or interstitial lung diseases^13^. MUC1 may also influence resistance to corticosteroid treatments, which is related to corticosteroid resistance in patients with asthma. In some patients, epigenetic changes could affect MUC1-CT and block its ability to inhibit inflammation, contributing to the progression of respiratory infections^13^.

There are several limitations to this study. The primary limitation is the absence of a serial measurement of CA 15-3 levels during admission; we recorded the concentrations on the day of admission, but the levels may vary depending on the stage of lung involvement at which they are measured. We think that it could aid in monitoring the patient´s condition, and increases in levels in the initial days could serve as a prognostic factor. Furthermore, we have not distinguished previous lung diseases presented by patients, which limits the conclusions drawn from the study because previous treatments could have influenced the evolution of the disease^45^.

## Conclusions

The degree of lung involvement is the main prognostic factor in SARS-CoV-2 infection. In the initial stages, the presence of silent hypoxemia may not reveal the severity of pulmonary involvement. SARS-CoV-2 infection has the effect of damaging the alveolar epithelium after the development of inflammatory processes that lead to ARDS and residual fibrotic changes. Biomarkers that express the degree of lung damage and fibrosis, such as CA 15-3, can help identify patients who will require a greater use of resources and could present a worse prognosis on admission.

## Supplementary Information

Supplementary Information.

## Data Availability

All data generated or analysed during this study are included in this published article [and its supplementary information files].

## Abbreviations

ARDS: Adult respiratory distress syndrome
CA15-3: Carbohydrate antigen 15-3
COPD: Chronic obstructive pulmonary disease
COVID-19: Coronavirus disease 2019
CRP: C-Reactive protein
CT: Cytoplasmic tail
KL-6: Krebs von den Lungen-6
OR: Odd ratio
PCR: Polymerase chain reaction
SARS-CoV-2: Coronavirus 2 severe acute respiratory syndrome
WHO: World Health Organization
