# Supplementary material for: CA 15-3 prognostic biomarker in SARS-CoV-2 pneumonia
Source: Sci Rep. 2022 Apr 25;12:6738. doi: 10.1038/s41598-022-10726-7 (PMC9037059; doi:10.1038/s41598-022-10726-7)
Supplement: Supplementary file 1 — Supplementary Information. [file 41598_2022_10726_MOESM1_ESM.docx]

**Table 1S**. Correlations between Ca15-3 concentrations and other risk factors for severe COVID19.

| **Variable** | **R** | **P** |
| --- | --- | --- |
| **Age** | 0,415 | < 0,001 |
| **D-dimer** | 0,281 | <0,001 |
| **Troponine** | 0,438 | <0,001 |
| **CRP** | 0,135 | 0,002 |
| **Ferritine** | 0,203 | <0,001 |
| **IL6** | 0,181 | <0,001 |
| **NT- ProBNP** | 0,301 | <0,001 |

**Table 2S**. Reference values of CA 15-3 for prediction of death.

|  | CA15-3 levels | Normals (n=465) | Abnormals (n=55) | SSLR (95% CI) | TPR | FPR | AUC (SE) |
| --- | --- | --- | --- | --- | --- | --- | --- |
| 5-stratum | **0 - 16.6** | 240 | 11 | 0.39 (0.23-0.65) | 0.80 | 0.48 | 0.73 (0.04) |
|  | **16.7 - 23.7** | 103 | 11 | 0.90 (0.53-1.55) | 0.60 | 0.26 |  |
|  | **23.8 - 34.4** | 95 | 11 | 0.98 (0.57-1.69) | 0.40 | 0.06 |  |
|  | **34.5 - 44.1** | 13 | 11 | 7.15 (3.42-14.93) | 0.20 | 0.03 |  |
|  | **44.2+** | 14 | 11 | 6.64 (3.23-13.68) | 0.00 | 0.00 |  |
| 3-stratum | **0 - 16.6** | 240 | 11 | 0.39 (0.23-0.65) | 0.80 | 0.48 | 0.73 (0.04) |
|  | **16.7 - 34.4** | 198 | 22 | 0.94 (0.67-1.31) | 0.40 | 0.06 |  |
|  | **34.5+** | 27 | 22 | 6.89 (4.25-11.16) | 0.00 | 0.00 |  |

**Table 3S**. Univariable logistic regression analysis of predictors at admission for in-hospital complications.

|  | **Death** | |  | **Intensive Care** | |  | **O_2_ at discharge** | |
| --- | --- | --- | --- | --- | --- | --- | --- | --- |
|  | OR (CI 95%) | p |  | OR (CI 95%) | p |  | OR (CI 95%) | p |
| **Age, per 10 years** | 1.94 (1.57, 2.44) | <0.001 |  | 1.29 (1.11, 1.51) | 0.001 |  | 1.60 (1.34, 1.95) | <0.001 |
| **Male** | 1.42 (0.81, 2.54) | 0.230 |  | 2.96 (1.74, 5.24) | <0.001 |  | 4.93 (2.60, 10.2) | <0.001 |
| **Respiratory disease** | 2.28 (1.19, 4.23) | 0.010 |  | 1.52 (0.82, 2.71) | 0.165 |  | 2.90 (1.55, 5.28) | <0.001 |
| **Smoking history** | 1.57 (0.84, 2.84) | 0.143 |  | 1.74 (1.02, 2.91) | 0.038 |  | 2.11 (1.20, 3.67) | 0.009 |
| **Hypertension** | 2.76 (1.56, 5.02) | <0.001 |  | 1.82 (1.12, 2.96) | 0.016 |  | 2.92 (1.71, 5.12) | <0.001 |
| **Diabetes** | 2.18 (1.22, 3.85) | 0.008 |  | 1.33 (0.78, 2.23) | 0.288 |  | 1.61 (0.90, 2.82) | 0.100 |
| **Cardiovascular disease** | 2.14 (1.11, 3.96) | 0.018 |  | 0.88 (0.43, 1.64) | 0.691 |  | 2.24 (1.18, 4.10) | 0.011 |
| **History of cancer** | 4.11 (1.96, 8.26) | <0.001 |  | 1.72 (0.78, 3.52) | 0.152 |  | 1.17 (0.39, 2.92) | 0.750 |
| **Length of symptoms, days** | 0.95 (0.88, 1.02) | 0.188 |  | 1.03 (0.98, 1.08) | 0.196 |  | 1.00 (0.95, 1.05) | 0.917 |
| **WHO scale, per unit** | 5.45 (3.39, 9.09) | <0.001 |  | 62.0 (26.1, 185) | <0.001 |  | 23.1 (10.5, 61.4) | <0.001 |
| **D-dimer, log10** | 5.87 (3.28, 10.8) | <0.001 |  | 2.77 (1.65, 4.67) | <0.001 |  | 1.72 (0.89, 3.22) | 0.095 |
| **Ferritine, log10** | 3.36 (1.78, 6.63) | <0.001 |  | 7.51 (4.00, 14.8) | <0.001 |  | 4.06 (2.18, 7.93) | <0.001 |
| **IL-6, log10** | 5.33 (3.02, 9.96) | <0.001 |  | 2.75 (1.75, 4.44) | <0.001 |  | 3.26 (1.87, 5.99) | <0.001 |
| **CRP, log10** | 4.13 (2.09, 8.79) | <0.001 |  | 3.53 (1.99, 6.60) | <0.001 |  | 2.94 (1.61, 5.69) | <0.001 |
| **TnThs, log10** | 13.7 (7.08, 28.0) | <0.001 |  | 2.90 (1.74, 4.88) | <0.001 |  | 5.04 (2.70, 9.59) | <0.001 |
| **NT-proBNP, log10** | 3.17 (2.25, 4.57) | <0.001 |  | 1.79 (1.34, 2.39) | <0.001 |  | 2.12 (1.53, 2.95) | <0.001 |
| **CA15-3** |  |  |  |  |  |  |  |  |
| **0 – 16.7, reference** | - | - |  | - | - |  | - | - |
| **16.7 – 34.5** | 2.42 (1.17, 5.30) | 0.020 |  | 1.92 (1.08, 3.47) | 0.028 |  | 1.38 (0.79, 2.43) | 0.263 |
| **cat+34.5** | 17.8 (7.96, 42.0) | <0.001 |  | 10.4 (5.13, 21.6) | <0.001 |  | 3.16 (1.21, 7.66) | 0.014 |

**Table 4S**. WHO COVID Ordinal Scale.

| **Patient State** | **Descriptor** | **Score** |
| --- | --- | --- |
| **Ambulatory** | No limitation of activities | 1 |
|  | Limitation of activities | 2 |
| **Hospitalized, mild disease** | No oxygen therapy | 3 |
|  | Oxygen by mask or nasal cannule | 4 |
| **Hospitalized, severe disease** | Non-invasive ventilation or high-flow oxygen | 5 |
|  | Invasive mechanical ventilation without other organ support | 6 |
|  | Invasive mechanical ventilation with other organ support.  ECMO | 7 |
| **Death** | Dead | 8 |
